# Genetic characterisation of the recent foot-and-mouth disease virus subtype A/IRN/2005

**DOI:** 10.1186/1743-422X-4-122

**Published:** 2007-11-15

**Authors:** Joern Klein, Manzoor Hussain, Munir Ahmad, Preben Normann, Muhammad Afzal, Soren Alexandersen

**Affiliations:** 1 National Veterinary Institute, Technical University of Denmark, Lindholm, DK-4771 Kalvehave, Denmark; 2 Food and Agriculture Organization of the United Nations – Pakistan, NARC, Park Road, PK-45500, Pakistan; 3 Ministry of Food, Agriculture & Livestock Pakistan, Livestock wing, PK-44000, Pakistan

## Abstract

**Background:**

According to the World Reference Laboratory for FMD, a new subtype of FMDV serotype A was detected in Iran in 2005. This subtype was designated A/IRN/2005, and rapidly spread throughout Iran and moved westwards into Saudi Arabia and Turkey where it was initially detected from August 2005 and subsequently caused major disease problems in the spring of 2006. The same subtype reached Jordan in 2007. As part of an ongoing project we have also detected this subtype in Pakistan with the first positive samples detected in April 2006.

To characterise this subtype in detail, we have sequenced and analysed the complete coding sequence of three subtype A/IRN/2005 isolates collected in Pakistan in 2006, the complete coding sequence of one subtype A/IRN/2005 isolate collected during the first outbreak in Turkey in 2005 and, in addition, the partial 1D coding sequence derived from 4 epithelium samples and 34 swab-samples from Asian buffaloes or cattle subsequently found to be infected with the A/IRN/2005 subtype.

**Results:**

The phylogenies of the genome regions encoding for the structural proteins, displayed, with the exception of 1A, distinct, serotype-specific clustering and an evolutionary relationship of the A/IRN/2005 sublineage with the A22 sublineage. Potential recombination events have been detected in parts of the genome region coding for the non-structural proteins of FMDV.

In addition, amino acid substitutions have been detected in the deduced VP1 protein sequence, potentially related to clinical or subclinical outcome of FMD.

Indications of differential susceptibility for developing a subclinical course of disease between Asian buffaloes and cattle have been detected.

Furthermore, hitherto unknown insertions of 2 amino acids before the second start codon, as well as sublineage specific amino acids have been detected in the genome region encoding for the leader proteinase of A/IRN/2005 sublineage.

**Conclusion:**

Our findings indicate that the A/IRN/2005 sublineage has undergone two different paths of evolution for the structural and non-structural genome regions.

The structural genome regions have had their evolutionary starting point in the A22 sublineage. It can be assumed that, due to the quasispecies structure of FMDV populations and the error-prone replication process, advantageous mutations in a changed environment have been fixed and lead to the occurrence of the new A/IRN/2005 sublineage.

Together with this mechanism, recombination within the non-structural genome regions, potentially modifying the virulence of the virus, may be involved in the success of this new sublineage.

The possible origin of this recombinant virus may be a co-infection with Asia1 and a serotype A precursor of the A/IRN/2005 sublineage potentially within Asian Buffaloes, as these appears to relatively easy become infected, but usually without developing clinical disease and consequently showing not a strong acute inflammatory immune response against a second FMDV infection.

## Background

Foot-and-mouth disease (FMD) is a highly communicable and economically important disease caused by foot-and-mouth disease virus (FMDV). Animals that can be affected include cattle, swine, sheep, goats, wild pigs, wild ruminants and buffaloes [1]. FMDV is a positive sense single-stranded RNA virus (genus *Aphthovirus*, family *Picornaviridae*) occurring in seven serotypes, O, A, C, Asia1, SAT 1, SAT 2 and SAT 3, each with a wide spectrum of antigenic and epidemiological different subtypes. The wide diversity is considered a consequence of the high mutation rate, quasi-species dynamics [2] and recombination [3,4].

Within the seven serotypes, serotype A displays the greatest number of newly occurring subtypes, which makes the control by vaccination very difficult [5].

During 2005, a new FMDV A subtype, A/IRN/2005, spread throughout Iran and moved westwards into Saudi Arabia, Turkey and in 2007 reached Jordan [6]. In 2006, we have also detected this subtype in Pakistan [7]. This particular FMDV subtype has proven to be highly virulent and has caused severe disease in all ages of cattle [8].

Serum neutralization assays demonstrated a closer relationship to A22 than to other serotype A subtypes [9] and the World Reference Laboratory as well as the FAO European Commission for the control of FMD recommend, in the absence of an homologous vaccine strain, the use of the widely available A22 Iraq strain as vaccine [6].

For an ongoing study in Landhi Cattle Colony (LCC), Pakistan, we have collected more than thousand swab-samples from randomly selected Asian buffaloes and cattle without clinical signs of FMD, as well as a number of epithelium samples from clinical FMD cases. Landhi cattle colony consists of approximately 2000 farms with a total population of approximately 300000 animals, of which 90% are Asian buffalos and furthermore a high number of free ranging sheep and goats. FMD vaccination is applied to a high degree in the cattle and buffalo population, using legally purchased and black market vaccines.

We have sequenced and analysed the complete coding sequence of three A/IRN/2005 isolates collected in Pakistan in 2006 and also the complete coding sequence of one isolate collected during the first outbreak in Turkey in 2005. In addition, we have analysed partial 1D sequences derived from 4 vesicular epithelium samples and from 34 mouth swabs collected in Pakistan from Asian buffaloes and cattle found subsequently infected with this subtype.

## Results

### Phylogenetic inference

The complete coding sequence (CDS) of four A/IRN/2005-like isolates, three originating from Karachi, Pakistan (Pakistan1, Pakistan3, Pakistan5) and collected in spring 2006, and in addition, the first recognized outbreak of A/IRN/2005 in Turkey in spring 2005, Turkey(WRL), have been compared with sequences published in Genbank (Table 1). Phylogenies have been inferred for the complete CDS, as well as for each protein coding genome-region (a schematic drawing of the FMDV genome is shown in Additional file 1).

**Table 1:** Selection of isolates used in this study

**accession-no.**	**serotype**	**isolate**	**year of isolation**	**published by**
EF117837	A	Pakistan3	2006	this study
EF494486	A	Turkey(WRL)	2005	this study
EF494487	A	Pakistan1	2006	this study
AY593791	A	Iran105	1998	[3]
EF494488	A	Pakistan5	2006	this study
AY593803	A	Venceslau	1976	[3]
AY593787	A	Bagge77	1977	[3]
AY593752	A12	Valle	1932	[3]
AY593755	A15	Thailand	1960	[3]
AY593756	A16	Belem	1959	[3]
AY593765	A22	Turkey	1965	[3]
AY593763	A22	Iraq64	1964	[3]
AY593764	A22	Iraq70	1970	[3]
AY593762	A22	Iraq95	1995	[3]
AY593763	A22	Iraq64	1964	[3]
AY593765	A22	Turkey66	1966	[3]
AY593766	A23	Kenya	1965	[3]
DQ767862	A	Iran	2006	unpublished
AY593772	A28	Turkey	1972	[3]
AY593800	Asia1	Leb83	1983	[3]
AY687333	Asia1	India01	2001	[13]
AY593798	Asia1	Leb89	1983	[3]
AY593799	Asia1	Leb4	1983	[3]
AY304994	Asia1	India63	vaccine	[3]
AY687334	Asia1	India97	vaccine	[3]
AY593807	C3	Resende	1955	[3]
DQ404179	O	UK	2001	[20]
DQ404168	O	UK	2001	[20]
DQ404180	O	UK	2001	[20]
EF611987	O	Uganda	2006	this study
AY593834	O	Iran	1966	[3]
DQ404163	O	UK	2001	[20]
AJ539138	O	CHA99	1999	[3]
AJ539137	O	TAW2	1999	[3]
AJ539140	O	SAR	1999	[3]
AJ539139	O	SKR	2000	[3]
AF377945	O	SKR	2000	[3]
AB079061	O	JPN	2000	[3]
AJ539136	O	TAW2	1999	[3]
AF506822	O	CHA	1999	[3]
AJ633821	O	FRA	2001	[3]
AY333431	O	NY	2000	[3]
AF506822	O	CHA	1999	[3]
AY593824	O1	SKR	2000	[3]
AF189157	O1	Geshure	unknown	[3]
AY593823	O1	Manisa	1969	[3]
AY593821	O1	Caseros	1967	[3]
AF283435	O5	India	2000	[21]
AY593828	O5	India	1962	[3]

**Figure 1:**
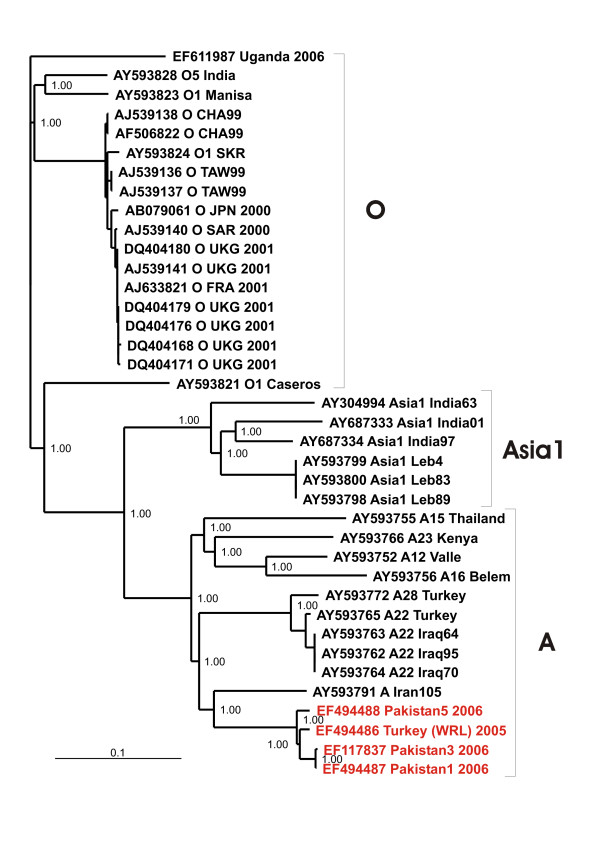
**Bayesian phylogenetic analysis of the complete coding sequence of the A/IRN/2005 sublineage (red) and related published sequences (black)**. Numbers on the nodes indicate clade credibility values.

Figure 1 displays the phylogeny of the complete CDS of the three serotypes O, Asia1 and A, showing the close relationship of the A/IRN/2005 subtype to the A22 and A28 subtypes, circulating in the Middle East region. The A/IRN/2005 subtype shares a common ancestor with A Iran105 which originated from Iran in 1998.

**Figure 2:**
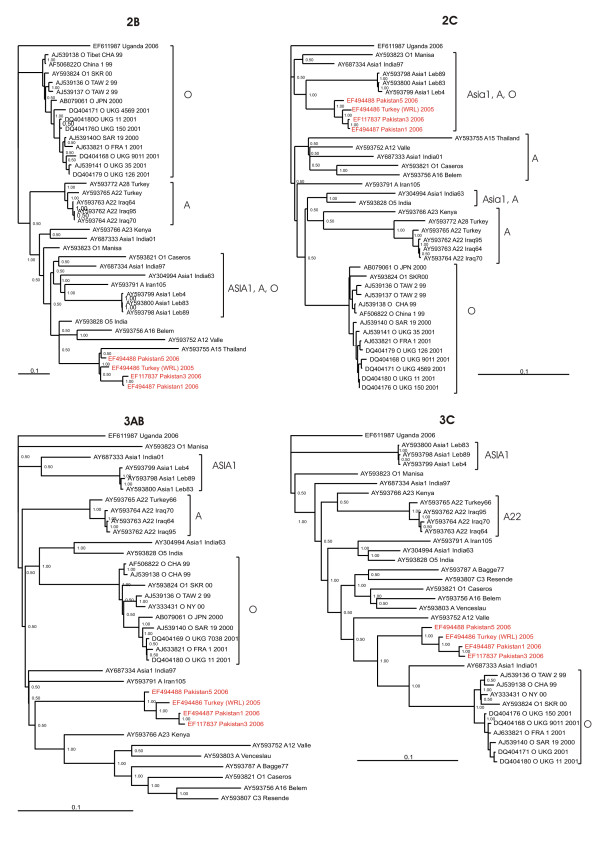
**Bayesian phylogenetic analysis of the genome regions 2B, 2C, 3AB and 3C, coding for non-structural proteins of the A/IRN/2005 sublineage (red) and related published sequences (black)**. Numbers on the nodes indicate clade credibility values.

**Figure 3:**
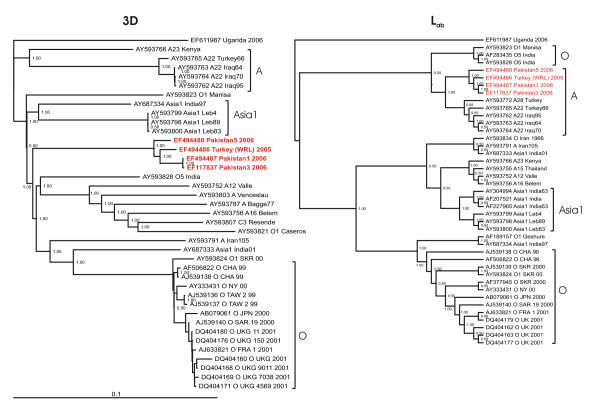
**Bayesian phylogenetic analysis of the genome regions 3D and L_ab_, coding for non-structural proteins of the A/IRN/2005 sublineage (red) and related published sequences (black)**. Numbers on the nodes indicate clade credibility values. The inferred phylogeny of the L_ab _genome region is, for better legibility, displayed as cladogram.

Figures 2 and 3 displays the inferred phylogenies of the genome regions coding for the nonstructural proteins. The phylogeny of 2B place the A/IRN/2005 sublineage in close relation to an A15 lineage from Thailand isolated in 1960 and in further relation to A16 Belem, isolated in 1959, A12 Valle, isolated in 1932 and O5 India, isolated in 1962. The inferred phylogeny of the 2C genome region displays a clear relationship between the A/IRN/2005 sublineage and an Asia1 lineage originating from the Lebanon, as well as an relation with the Indian vaccine strain for Asia1 India97 and O1 Manisa. In both phylogenies non-serotype specific grouping can be seen between some Asia1, A and O sublineages, however the PanAsia sublineage of serotype O and the A22, A23 and A28 sublineage of serotype A are monophyletic, i.e. it consists of an inferred common ancestor. The latter sublineages are well separated from A/IRN/2005.

The phylogeny of the coding sequence of the 3AB nonstructural proteins, which are important for RNA replication, displays the A/IRN/2005 sublineage as a monophyletic group, but with an relation to a group of at least 31 years old isolates, consisting of serotypes O, A and C. Members of the latter are also in relation to A/IRN/2005 sublineage within the 2B phylogeny.

Also the phylogeny of the coding sequence for the 3C protease, present the A/IRN/2005 sublineage as a monophyletic group, sharing a common ancestor with the PanAsia sublineage of serotype O and the Indian vaccine strain for Asia1 India97. The 3C phylogeny shows a number of non-serotype specific clustering.

The phylogeny of the coding sequence for the RNA-dependent RNA polymerase 3D, displays the A/IRN/2005 sublineage as an monophyletic group, sharing at one point of time a common ancestor with the Panasia lineage of serotype O and again with the previously mentioned group of at least 31 years old isolates, as well as with the A Iran105 and Asia1 India01 isolates.

The cladogram of the complete Leader protease coding region present the A/IRN/2005 sublineage most related to the A22/A28 lineages, but still with a clear evolutionary distance (see Additional file 2).

The phylogenies of the genome regions encoding for the structural proteins (see Additional file 3), display, with the exception of 1A, distinct, serotype-specific clustering.

**Figure 4:**
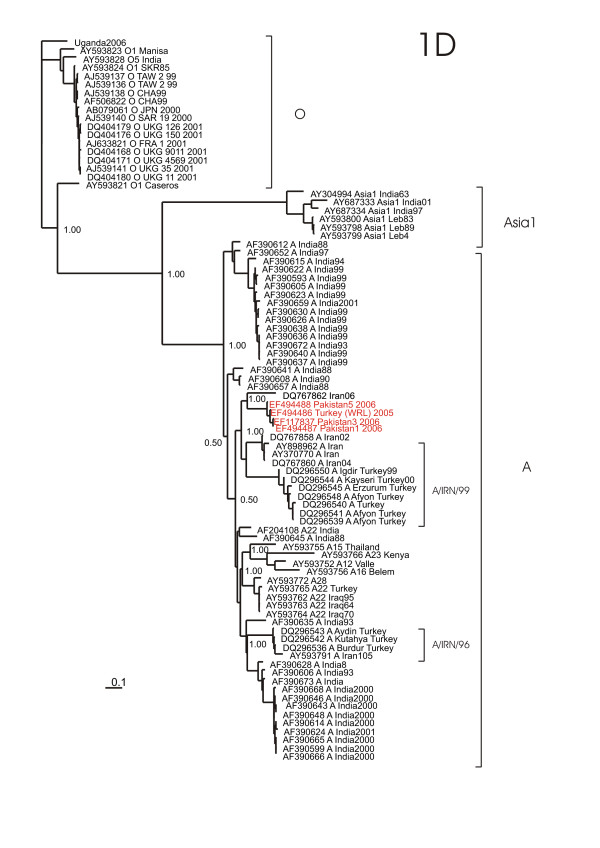
**Bayesian phylogenetic analysis of the 1D genome region of the A/IRN/2005 sublineage (red) and related published sequences (black)**. Numbers on the nodes indicate clade credibility values.

The A/IRN/2005 sublineage shares a common ancestor with A Iran105, isolated during 1998 in Iran, within the phylogenies for 1A to 1C. In these inferred phylogenies the A/IRN/2005 sublineage is also in close relationship to the A22 lineage. However, the phylogenetic analysis of the 1D genome region (Figure 4), encoding for the VP1 protein, shows that the A/IRN/2005 sublineage clusters together with the A/IRN/99 sublineage, whereas A Iran105 clusters together with the A/IRN/96 sublineage. The A22 sublineage is here well separated from the A/IRN/2005 sublineage.

### Amino acid comparison of the partial VP1 surface protein

**Figure 5:**
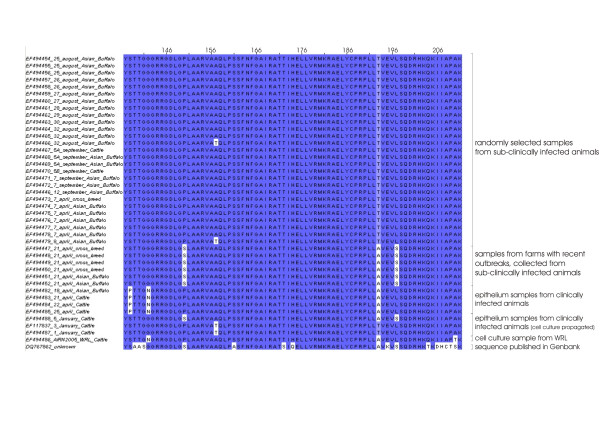
Alignment of the deduced amino acid sequences of the immuno-dominant residues of the VP1 surface protein, including the GH-loop.

Figure 5 shows the alignment of the deduced amino acid sequences of the immuno-dominant residues of the VP1 surface protein, including the GH-loop. The alignment consist only of isolates belonging to the FMDV A/IRN/2005 sublineage, collected in Karachi, Southern Pakistan (with exception of Turkey (WRL) and an Iranian isolate from 2006, DQ767862), but with three different sampling strategies. The first group consists of samples from animals which showed no indication of acute FMD; those animals are randomly selected from randomly selected herds. If, during this sampling, animals with healing FMD lesions were detected, then those samples were assembled to group 2, subclinically infected animals, with a recent outbreak history on the farm. The last group consists of targeted collected epithelium samples from animals with acute FMD.

All isolates, except four (Pakistan1, Pakistan3, Pakistan5 and Turkey (WRL)), have been directly sequenced, without cell culture propagation of the virus.

Out of the group of randomly selected isolates from sub-clinically infected animals it can be seen that all have the integrin binding motif 'RGDLGPL' and threonine at residue 193 in common. Sequence information from isolates collected from a farm with recent (1–2 weeks) clinical outbreak, but samples from subclinically infected animals, displayed the integrin binding motif 'RGDLGSL' and an alanine at residue 193, as well as a serine at residue 197 in common. Sequences from clinical affected animals displayed a proline at residue 138 and asparagine at residue 142, as well as alanine at position 193.

Of those isolates, where the virus has been propagated in cell culture two display alanine at residue 57 and one (EF494488 Pakistan5) a 'RGDLGSL' integrin binding motif, whereas the others in this group have an 'RGDLGPL' motif.

### Amino acid comparison of the Leader protease

**Figure 6:**
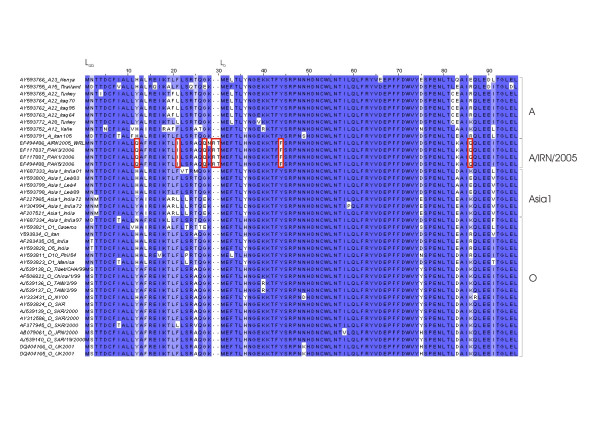
**Alignment of the deduced amino acid sequence of the first 96 residues of the FMDV Leader protease**. A/IRN/2005 specific residues are marked with a red box.

Figure 6 displays the alignment of the deduced amino acid sequence of the first 96 residues of the FMDV Leader protease from the A/IRN/05 sublineage together with published sequences. The alignment shows a serotype-specific distribution (see also Figure 3), however the A/IRN/2005 sublineage is unique with an insertion of two amino acids, arginine and threonine, before the second start codon, and a phenylalanine, instead of tyrosine, at residue 44. The sublineage shows also specific amino acids at residues 13, 21, 27 and 86, namely glutamine, isoleucine, glutamine and glutamine.

### Virulence and Host species

Out of nine isolates, collected from clinically affected animals, seven (78%) have been derived from cattle (*Bos taurus*) and two (22%) from Asian buffalo (*Bubalus bubalis*). In contrast twenty-four (89%) out of twenty-seven isolates from sub-clinically infected animals, originate from Asian Buffalo. This means that the proportion of clinically affected animals in the bovine species is 0.7 and in buffalo is 0.1. By comparing these proportions with each other and applying a two-sample t-test, the t-statistic was significant at the 0.05 critical alpha level, t(96) = 3.134, p = 0.0023. Therefore, it can be concluded that the difference in the proportion of clinically affected animals in the bovine species and in Asian Buffalo is significant. This difference is also supported by personal observation by the authors, as well as the experience of the local veterinarians.

## Discussion

A major question within the epidemiology of FMDV serotype A is why so many new lineages so regularly appear and why there is such a great antigenic diversity within this serotype [5]. Frequent recombinations [10], as well as long time circulation in poorly monitored areas and species [5], have been mentioned as an explanation for this characteristic.

This work shows that both factors may have contributed to the appearance of sublineage A/IRN/2005.

The overall evolutionary development, mirrored by the phylogeny of the complete CDS of the A/IRN/2005 sublineage and published FMDV sequences (Figure 1) shows that the A/IRN/2005 sublineage is related to the A22 sublineage and the phylogeny shows also an evolutionary intermediate, namely A IRN105 isolated during 1998 in Iran. However, the 1D phylogeny places the A IRN105 in close relationship to the IRN96 sublineage, while the A/IRN/2005 sublineage is more related to the IRN99 sublineage (Figure 4). Both, IRN96 and IRN99, have caused outbreaks in recent years in Turkey and Iran [11]. This different placing may be explained by the higher phylogenetic resolution of the 1D region, due to the fact that there are much more sequences from this region published, than from other genome regions.

From these findings it can be inferred that the genome regions encoding for the structural proteins may have evolved from the A22 lineage, with different intermediates like A Iran105 and the A/IRN/99 sublineage, due to antigenic drift.

Jackson et al. [12] concluded that recombination between serotypes is probably widespread throughout the non-structural gene regions and this can also be shown for A/IRN/2005.

The evolutionary relationship of the A/IRN/2005 sublineage within the 2B and 3AB genome region and a group of at least 31 years old isolates is noticeable, but also that there are sharing a common ancestor with the Asia1 vaccine lineage India97 [13] and in the case of the 2B phylogeny also with other Asia1 isolates. Keeping in mind that the use of doubtfully produced and distributed vaccine is not uncommon in Pakistan and India, the possibility of introduction of old virus strains to the susceptible, vaccinated population is given, providing that vaccine viruses are not properly inactivated and thereby increasing the risk of recombination.

Within the phylogeny of the 2C genome region the A/IRN/2005 sublineage show a clear relation to Asia1 isolates from the Middle East, indicating recombination between Asia1 and the A/IRN/2005 sublineage.

The phylogeny of the 3D genome region demonstrates that the A/IRN/2005 sublineage shares a common ancestor with the same group of at least 31 years old isolates as in the phylogenies of 2B and 3AB and in addition with the Panasia lineage of type O, an Asia1 and O5 isolate from India and the A Iran105 isolate from Iran. A relationship between Asia1 and the Panasia lineage of serotype O has been shown previously [10].

The cladogram of the L_ab _coding region of the A/IRN/2005 sublineage constitute a close affinity to the A22 sublineage, however the A/IRN/2005 sublineage is still unique by displaying an hitherto unknown insertion of 2 amino acids before the second start codon, as well as sublineage specific amino acids (Figure 6). Remarkably, the L_ab _coding region of A Iran105 cluster together with the Asia1 isolate India01.

It has to be considered that the drawback, of this and many other molecular epidemiological studies, is that there is not a complete temporal and spatial covering of sequences available. However, our results give a strong evidence for potential recombination events in the nonstructural genome regions.

An additional finding of this study is that the majority of subclinical infections caused by the A/IRN/2005 sublineage occur within Asian Buffaloes. Considering that subclinical infections causes only weak inflammatory immune reactions [14], the probability of a subsequent infection of the same animal with another FMDV serotype, e.g. Asia1, and thereby the likelihood of inter-serotype recombination is increased.

Furthermore, the alignment of the deduced amino acid sequence of the highly variable part of the immuno-dominant part of the VP1 surface protein shows distinct patterns for samples originating from subclinically infected animals and clinically infected animals or with a recent outbreak of clinical infected animals. The VP1 protein plays a major role in virus cell entry and it has been shown that residue 193 (Figure 5) plays a role in heparan sulphate recognition [15], which is required for efficient infection of cells in culture [12] and it can be seen in Figure 5 that all, but two, of the sequences derived from clinical cases or from farms with a recent outbreak display an alanine instead of an threonine at this position. Within sequences derived from clinical cases a substitution of serine with proline can be seen at residue 138 and vice versa at residue 149, within the integrin binding motif. Unlike any of the other common amino acids, proline has a cyclic ring and its presence creates a fixed kink in a protein chain, leading to a change in the secondary structure and this change may also play a role in the virus attachment efficiency.

The scattered substitutions within the cell culture propagated isolates may be explained by adaptation processes in the absence of immune response [16].

It can be argued that those substitutions, which are likely going together with changes outside VP1, lead to the observed different susceptibility to develop FMD with clinical outcome between Asian Buffalo and Cattle hosts.

## Conclusion

Our findings indicate that the A/IRN/2005 sublineage has undergone two different paths of evolution for the structural and non-structural genome regions.

The structural genome regions have had their evolutionary starting point in the A22 sublineage, a long known and widely occurring lineage, with the AY593791 A IRN105, A/IRN/96 and A/IRN/99 sublineages as evolutionary intermediates. It can be assumed that, due to the quasispecies structure of FMDV populations and the error-prone replication process, advantageous mutations in a changed environment have been fixed and lead to the occurrence of the new A/IRN/2005 sublineage.

Together with this mechanism, recombination within the non-structural genome regions, potentially modifying the virulence of the virus, may be involved in the success of this new sublineage.

The possible origin of this recombinant virus may be a co-infection with Asia1 and a serotype A precursor of the A/IRN/2005 sublineage within the Asian Buffaloes. Related to this, the role of doubtful FMD vaccines have to be investigated.

It is likely that the new A/IRN/2005 sublineage persists or low- level circulates subclinically within the Asian Buffaloes and thereby the further opportunity for both accumulation of genetic variation and recombination due to multiple infections by different serotypes is given.

## Methods

### Virus isolates

Virus isolates have been collected as a part of a larger study and the sampling strategy is described there [7].

Nine mouth epithelium samples (EF494480, EF494481, EF494482, EF494483, EF494484, EF494485, EF494487, EF494488, EF494437) have been collected in Karachi, South Pakistan. FMDV from three of these epithelium samples (EF494487, EF494488, EF494437) have been propagated in primary cultures of calf kidney cells for three passages, the other six were directly sequenced from the sample. In addition, we collected thirty-four mouth swabs from subclinically infected and animals (EF494446 – EF494479). The IAH, Pirbright, provided us with cell culture propagated virus from the first recognized outbreak in Turkey (Turkey (WRL)).

### RNA extraction, reverse transcriptase – PCR and cycle sequencing

Tissue (100–150 mg) was homogenized in 1 ml RNA*pro*™ Solution (Qbiogene, USA) in a Lysing Matrix D tube (Qbiogene, Inc., USA) using a FP 120 Fast Prep™ Cell Disruptor (Qbiogene, USA). Total RNA was extracted using RNeasy-Mini Kit™ (Qiagen, Germany) according to the manufacturer's instructions. Total RNA from mouth swabs and cell culture propagated virus was extracted using QIAamp RNA Blood Mini Kit™ (Qiagen, Germany).

cDNA synthesis was done using Ready-To-Go™ You-Prime First-Strand Beads (GE Healthcare Life Sciences, Sweden), employing the primers NV27T and random hexamers pdN_6_.

Five μl of the template cDNA were added to 45 *μ*l of the PCR reaction mixture containing 0.2 μM primers (see Additional file 4), 200 μM each of dATP, dCTP, dGTP and dTTP, 10 mM Tris-HCl (pH 8.3), 50 mM KCl, 1.5 mM MgCl_2 _and 1 U of AmpliTaq^® ^Gold DNA polymerase (Applied Biosystems, UK). DNA was amplified with a DNA Thermal Cycler PE9700 (Perkin Elmer) by a two-step cycling reaction as follows: 95°C for 15 min, and five cycles of 94°C for 30 sec, 59°C for 2 min and 72°C for 30 sec, and then 35 cycles of 94°C for 30 sec, 61°C for 30 sec and 72°C for 30 sec, followed by a final extension step of 72°C for 10 min.

The resulting PCR products were examined by electrophoresis, using a 1,2% agarose gel, with a separation time of 1.5 hours at 6.5 V/cm.

Amplicons were visualised with ethidium bromide and subsequently extracted and purified from the agarose gel with QIAquick Gel Extraction kit (Qiagen, Germany). Cycle-sequencing of the overlapping amplicons was then performed by Agowa GmbH, Germany.

### Multiple alignment

Sequence assembling was performed with ContigExpress (VectorNTI^©^-software) and multiple alignment was performed by log-expectation comparison, using the MUSCLE (v.3.6) software [17].

### Phylogenetic analysis

Models of evolution were determined by hierarchical Likelihood-Ratio test of 24 substitution models, using the programs PAUP*(v. 10) and MrModeltest (v. 2.2) [18].

The GTR+G was used and Bayesian analysis was performed using MrBayes (v3.2) [19] with the following settings. The maximum likelihood model employed 6 substitution types ("nst = 6"), with base frequencies set to variable values ("statefreqpr = dirichlet(1,1,1,1)"). Rate variation across sites was modeled using a gamma distribution (rates = "invgamma"). The Markov chain Monte Carlo search was run with 4 chains for 500000 generations, with trees being sampled every 100 generations (the first 1000 trees were discarded as "burnin").

## Authors' contributions

JK participated in planning of the study and carried out the molecular epidemiological analysis and drafted the manuscript. MH and MA helped collecting the field isolates and delivered background information. PN helped in finding primers and optimizing the PCR. SA is project coordinator and conceived the study and helped to draft the manuscript. All authors read and approved the final manuscript.

## Supplementary Material

Additional file 1 Schematic drawing of the FMDV genome. Shows a schematic drawing of the FMDV genome. Click here for file

Additional file 2 Phylogram of of the L_ab _genome region. Shows the Phylogram of of the L_ab _genome region. Click here for file

Additional file 3 Bayesian phylogenetic analysis of the genome regions coding for the structural proteins of the A/IRN/2005 sublineage and related published sequences. Represents the phylogentic analysis of the FMDV genome regions 1A, 1B and 1C. Click here for file

Additional file 4 Primers used for this study. Shows the used PCR primers. Click here for file
