# Supplementary figures and images for: Genetic characterisation of the recent foot-and-mouth disease virus subtype A/IRN/2005
Source: Virol J. 2007 Nov 15;4:122. doi: 10.1186/1743-422X-4-122 (PMC2194681; doi:10.1186/1743-422X-4-122)

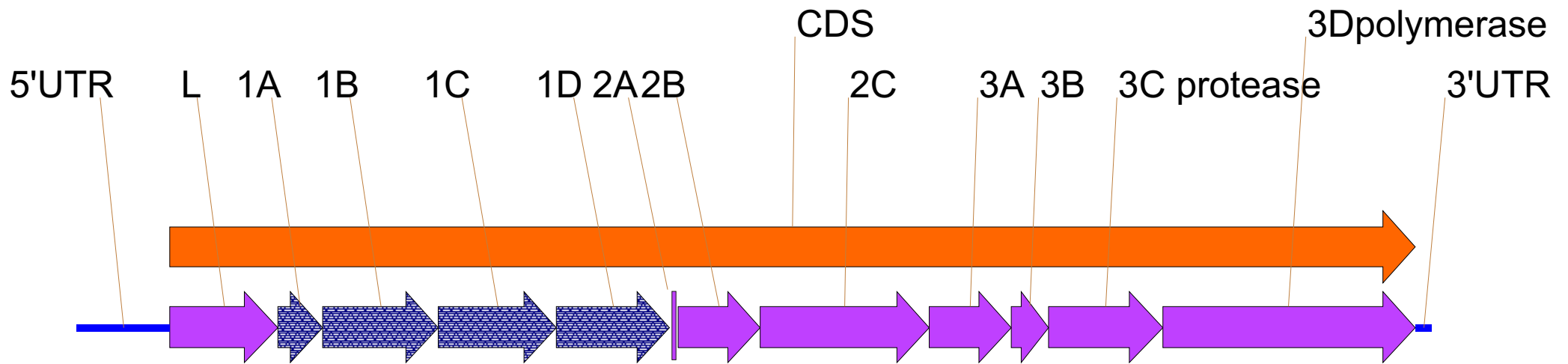

→ coding for nonstructural proteins

→ coding for structural proteins

Supplement: Additional file 1 — Schematic drawing of the FMDV genome. Shows a schematic drawing of the FMDV genome. [file 1743-422X-4-122-S1.pdf]

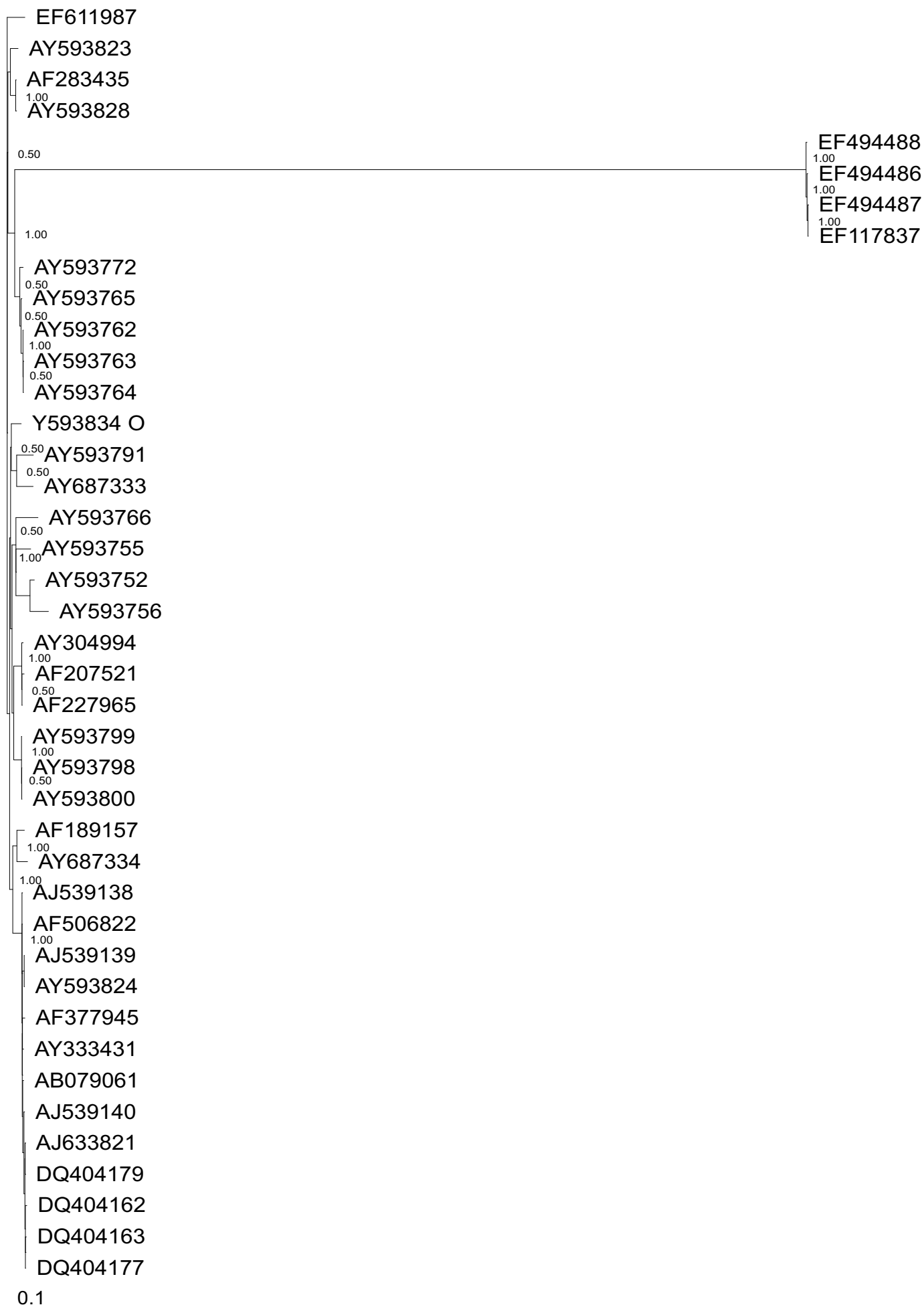

Supplement: Additional file 2 — Phylogram of of the Lab genome region. Shows the Phylogram of of the Lab genome region. [file 1743-422X-4-122-S2.pdf]

1A

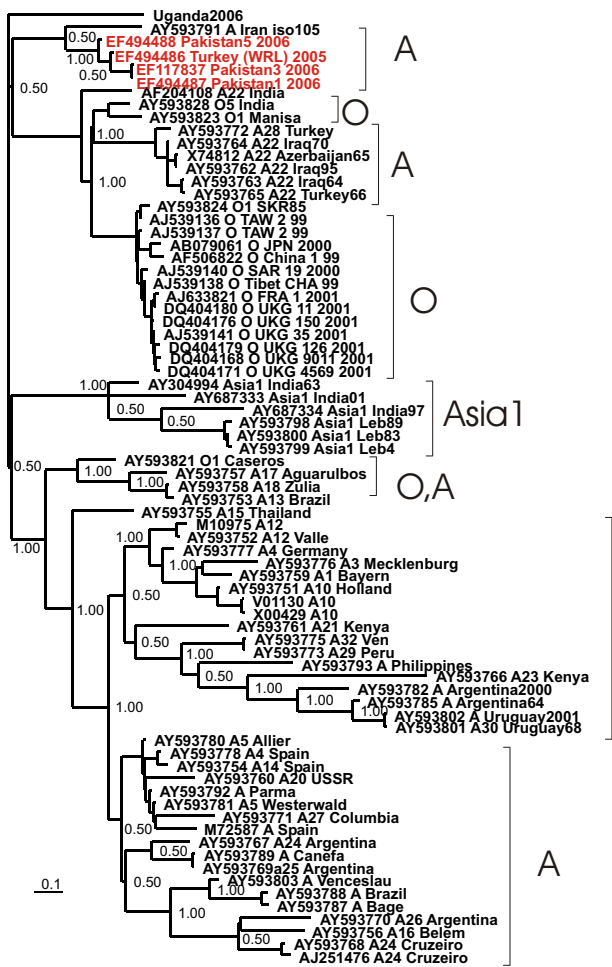

1B

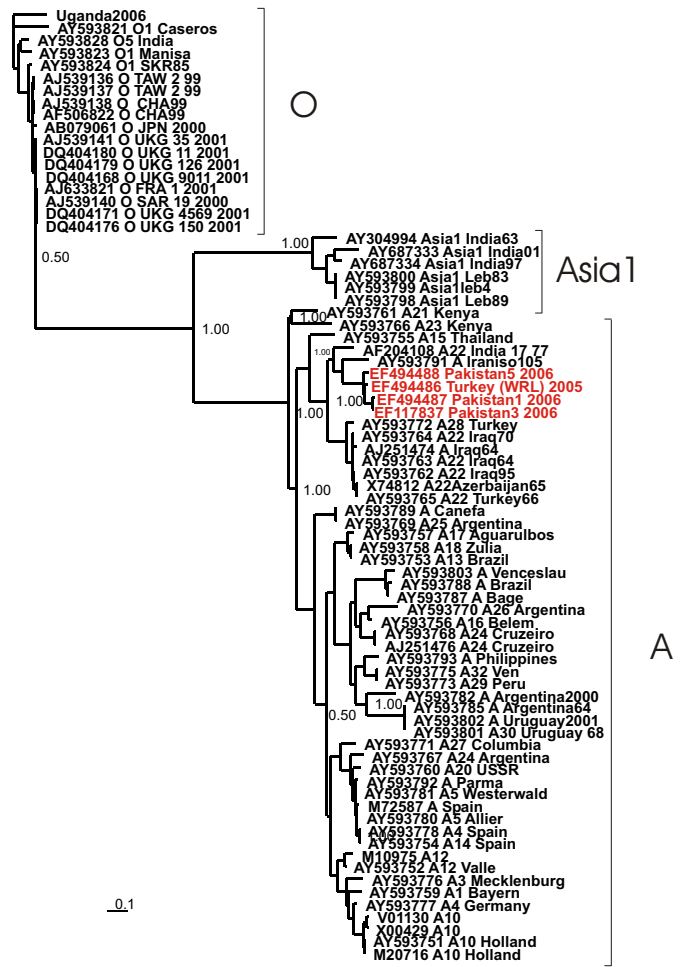

1C

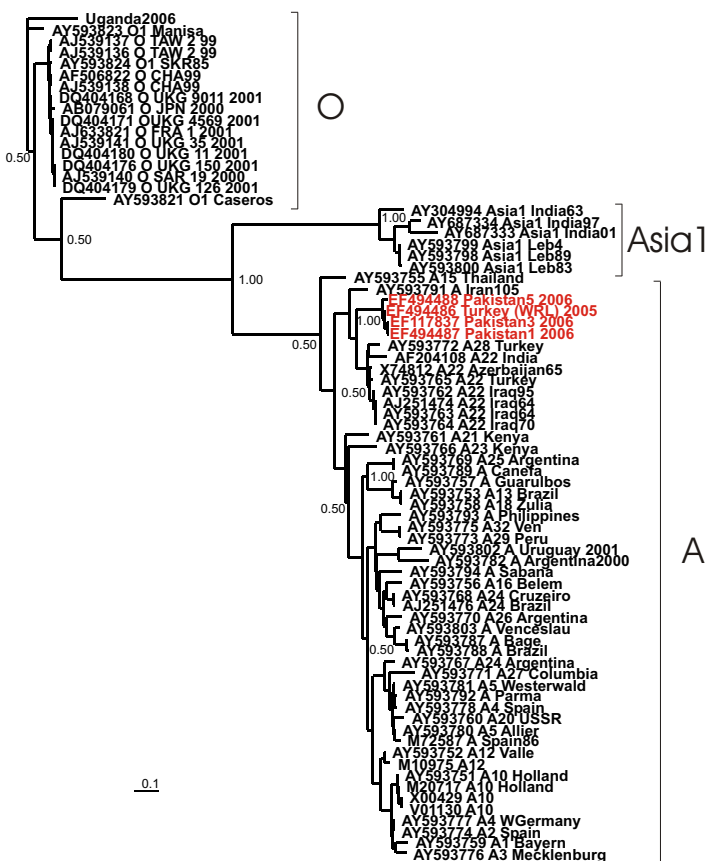

Supplement: Additional file 3 — Bayesian phylogenetic analysis of the genome regions coding for the structural proteins of the A/IRN/2005 sublineage and related published sequences. Represents the phylogentic analysis of the FMDV genome regions 1A, 1B and 1C. [file 1743-422X-4-122-S3.pdf]
