# Supplementary material for: Genetic characterisation of the recent foot-and-mouth disease virus subtype A/IRN/2005
Source: Virol J. 2007 Nov 15;4:122. doi: 10.1186/1743-422X-4-122 (PMC2194681; doi:10.1186/1743-422X-4-122)
Supplement: Additional file 4 — Primers used for this study. Shows the used PCR primers. [file 1743-422X-4-122-S4.pdf]

Primer name	Forward primer	Reverse primer
8-A PN 35	5´- GAGAAAIGGGACGTCIGCGC - 3´	
8-A PN 2		5´- CTTCTAIGCCTGAATAGGIGAC - 3´
10-P PN 23	5´- CGCCGTCGCTTGAGGAAGACTT - 3´	
10-P PN 24		5´- TTCTTTCGCGAGCACAGCAG - 3´
10-P PN 25	5´- TGCATGGTCGACGGGACAGA -3´	
10-P PN 26		5´- CATGAGAAATGGCTGGGACG - 3
8-A PN 51	5´- CCACAGATCAAGGTGTATGC - 3´	
8-A PN 6		5´- AACCTGCAITTCATGTIIACIGG - 3´
8-X PN 8	5´- CACATGTCAAACACTTACCTC - 3´	
10-P PN 11		5´- GACGTGGGTGCCCAATGG - 3´
10-P PN 27	5´- GGGGAAGACCATGTCTCCGGACCTA - 3´	
10-P PN 28		5´- AGGCTTGCCCGACCTTCCTCTGTT - 3´
10-P PN 29	5´- GCGTGCAGCCACGTACTACTTCT - 3´	
10-P PN 30		5´- TCTGGACAGCACCTTTGTCG - 3´
8-A PN 22	5´- AAGGACCCIGTCCTTGTGGC - 3´	
8-A PN 23		5´- GACCCTGACCACTTIGACGG - 3´
8-A PN 46	5´- TGGTCGTTTGCCTCCGTGG - 3´	
8-A PN 87		5´- GCAGCAATTGAATTCTTTGAG - 3´
8-A PN 99	5 - TGTACCAICTTGTTIAIGAGGTG - 3´	
8-A PN 68		5´- CCACCAGCTGAAGGACCC - 3´
8-A PN 113	5´- CGCGAIACTCGCAAGAGAC - 3´	
8-A PN 14		5´- CAGAGTGTTTGAGTTIGAGAT - 3´

8-A PN 207	5´- CTGGAGTGTTTGGCACTGC - 3´	
10-P PN 4		5´- CCAAGCTTGCACCCACCG - 3´
10-P PN 12	5´- CCACACCACGAGGGGTTG - 3´	
10-P PN 13		5´- GCGGTCGGTTGTAATCAA - 3´
10-P PN 14	5´- CTGAAGGACGAAATCCGC - 3´	
8-A PN 52		5´- GGTTTTGTTCTTGGTCAITCC - 3´
1-A TBR-1E	5´- GAGCTGGACACTTACACCATGATCTC - 3´	
NVT24		5´- AAAAAAAAAAAAAAAAAAAAAAAABN - 3´
